# Supplementary figures and images for: Deep brain stimulation of the Tbr1-deficient mouse model of autism spectrum disorder at the basolateral amygdala alters amygdalar connectivity, whole-brain synchronization, and social behaviors
Source: PLoS Biol. 2024 Jul 16;22(7):e3002646. doi: 10.1371/journal.pbio.3002646 (PMC11280143; doi:10.1371/journal.pbio.3002646)

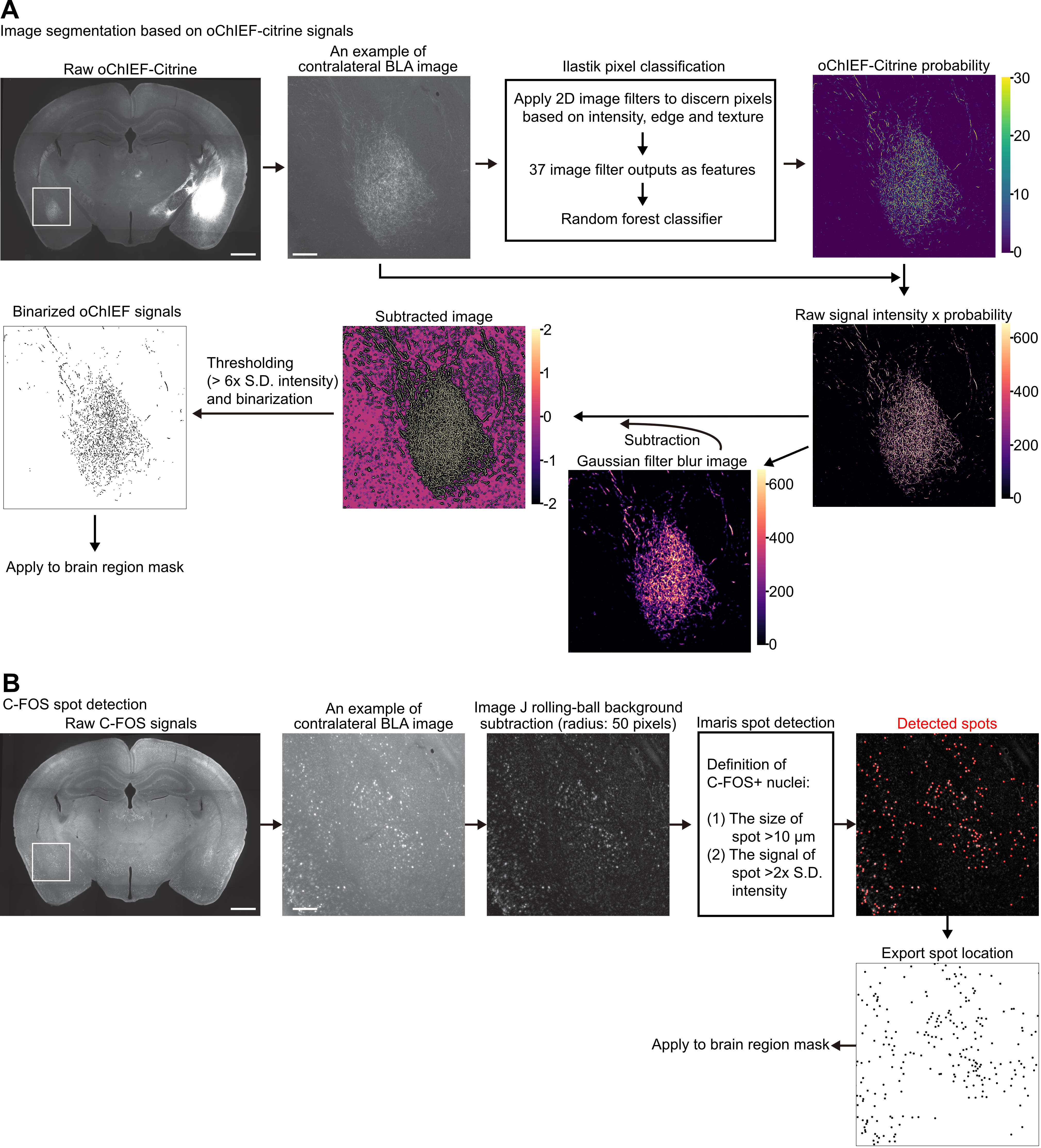

Supplement: S1 Fig — (JPG) [file pbio.3002646.s001.jpg]

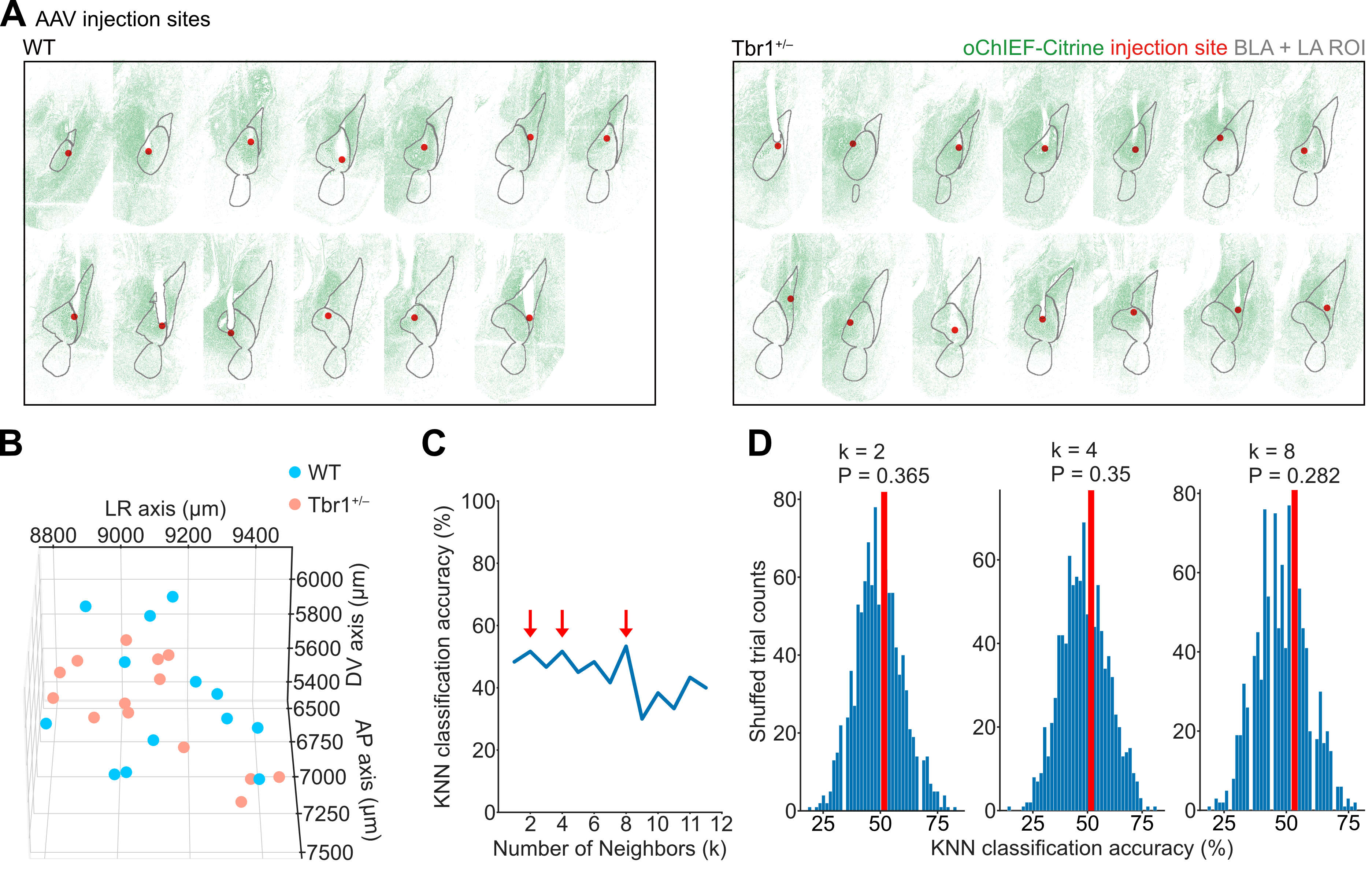

Supplement: S2 Fig — (JPG) [file pbio.3002646.s002.jpg]

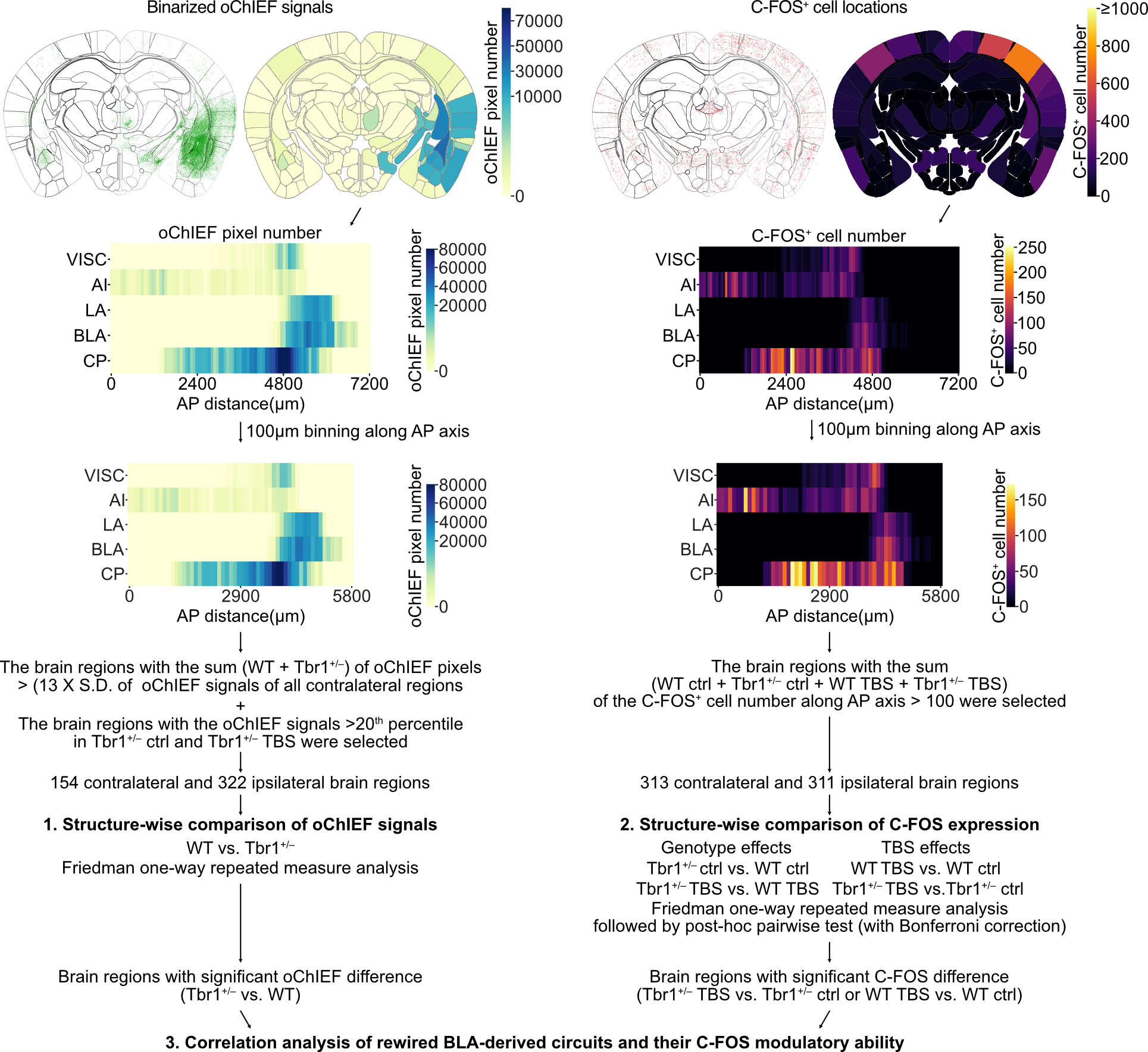

Supplement: S3 Fig — (JPG) [file pbio.3002646.s003.jpg]

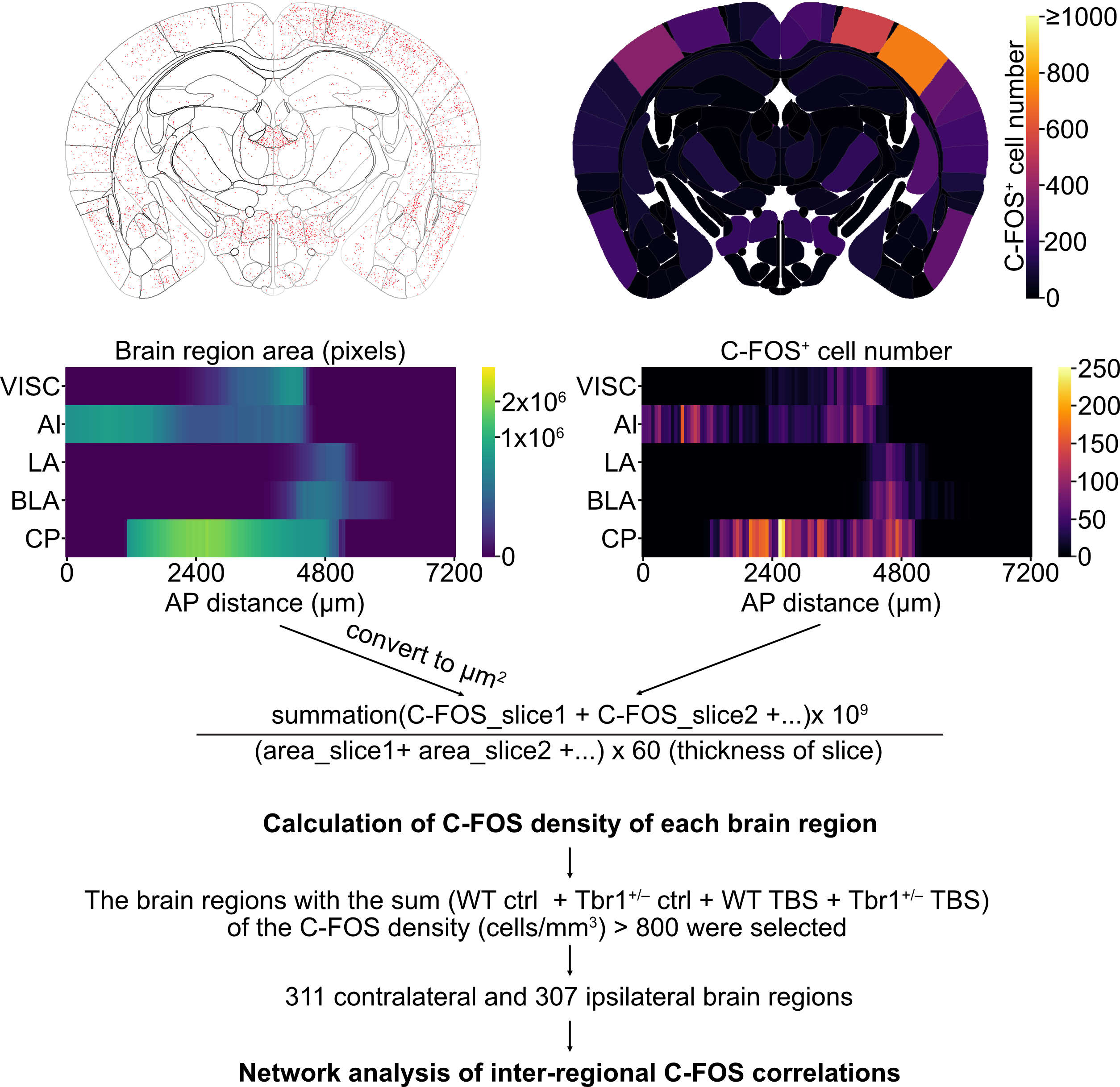

Supplement: S4 Fig — (JPG) [file pbio.3002646.s004.jpg]

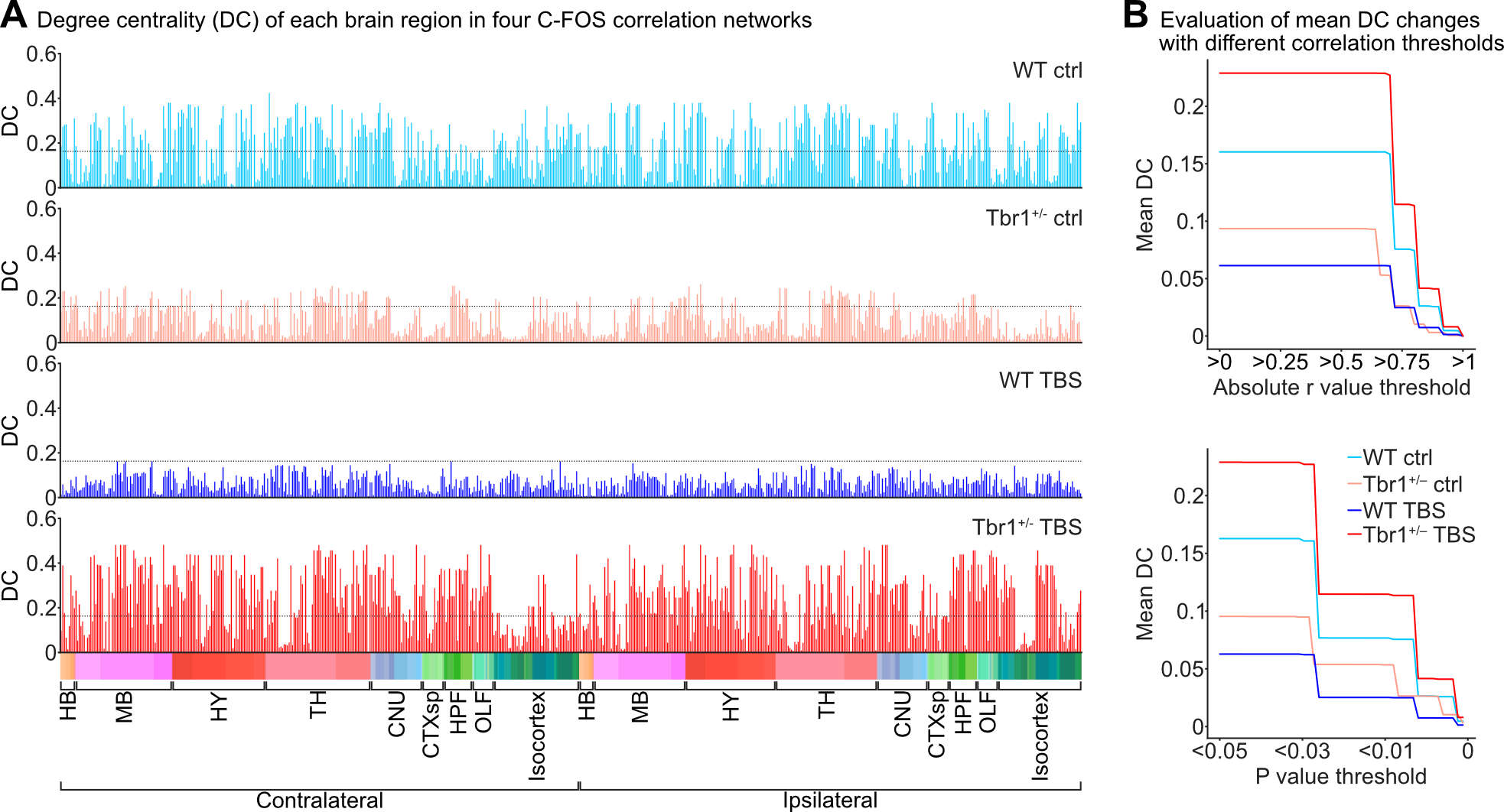

Supplement: S5 Fig — (JPG) [file pbio.3002646.s005.jpg]

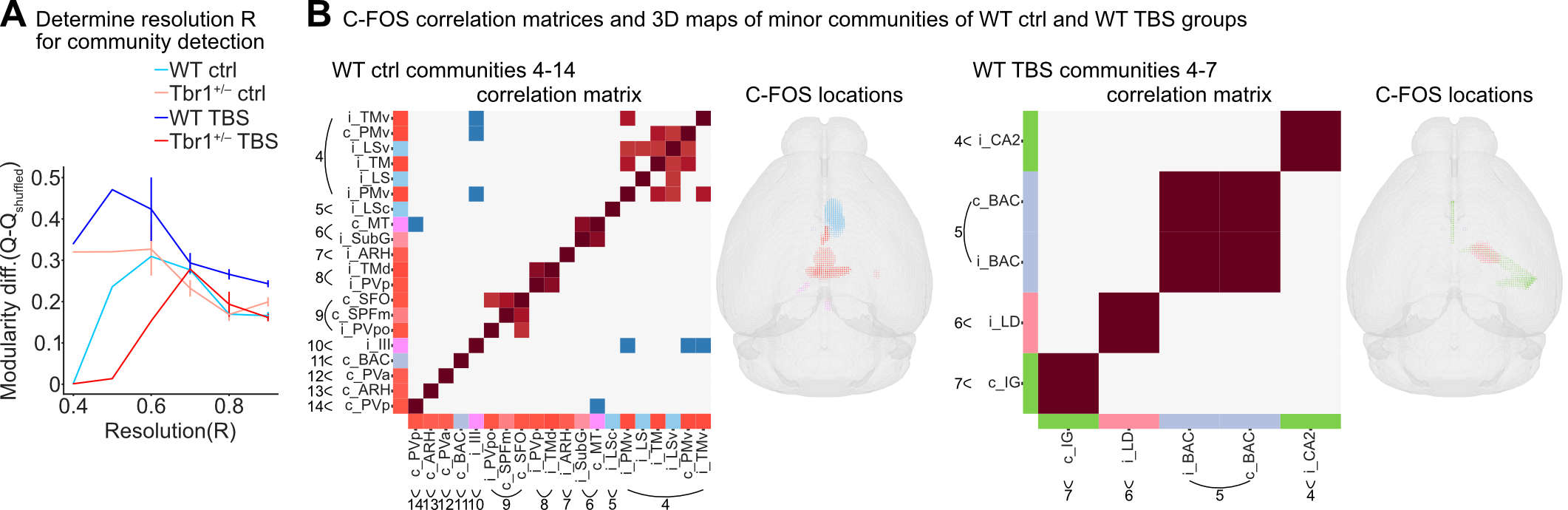

Supplement: S6 Fig — (JPG) [file pbio.3002646.s006.jpg]

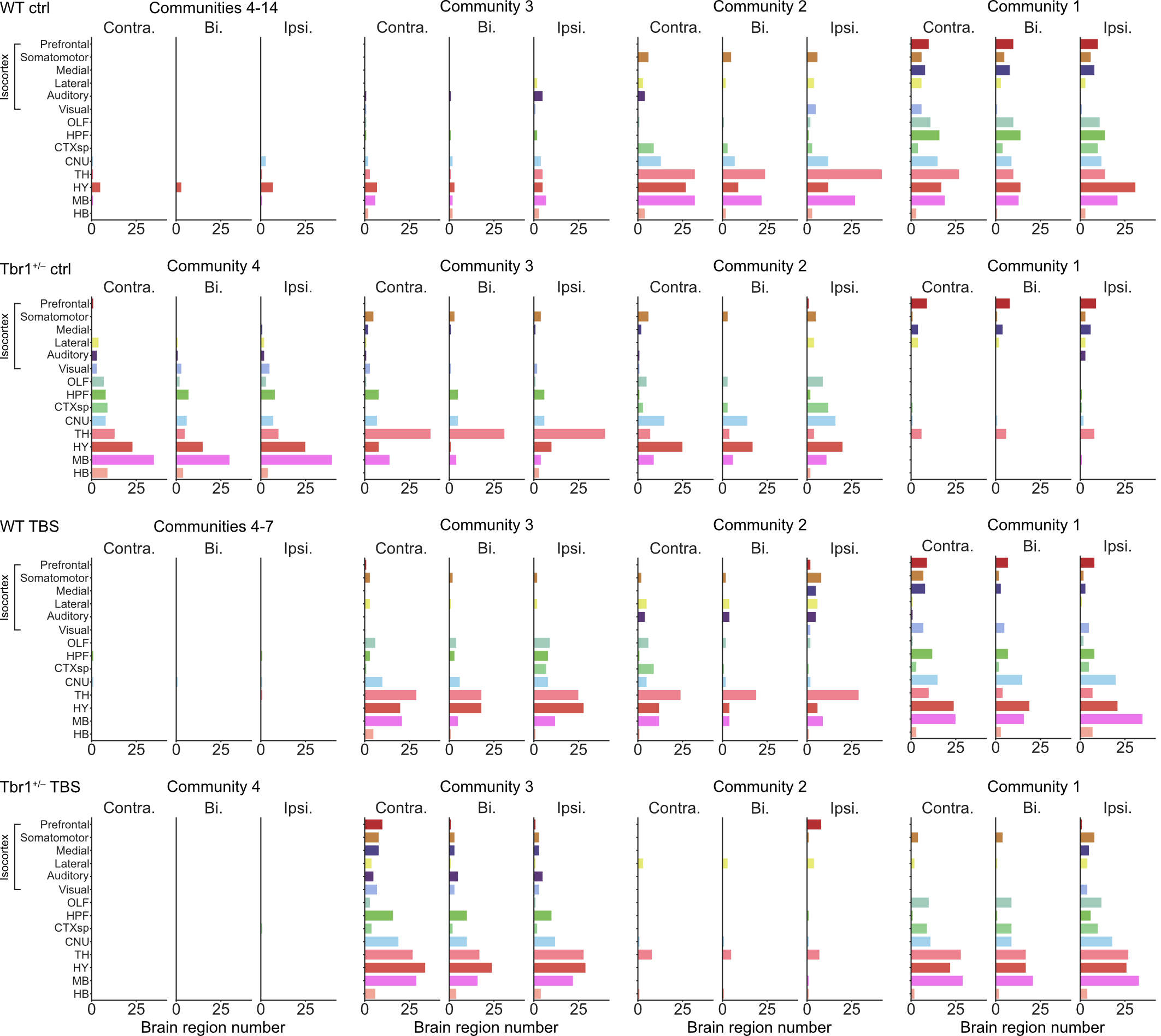

Supplement: S7 Fig — (JPG) [file pbio.3002646.s007.jpg]

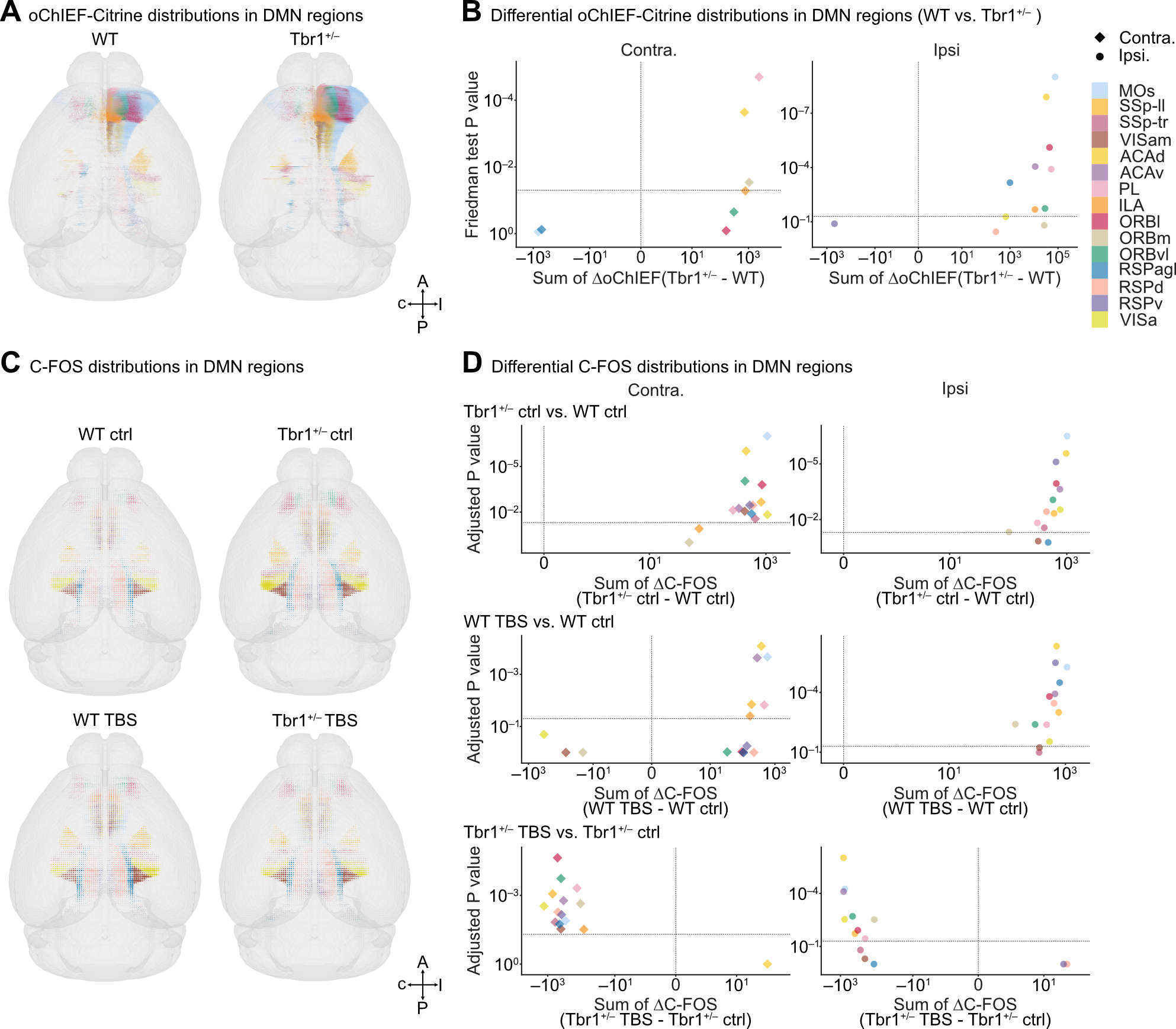

Supplement: S8 Fig — (JPG) [file pbio.3002646.s008.jpg]

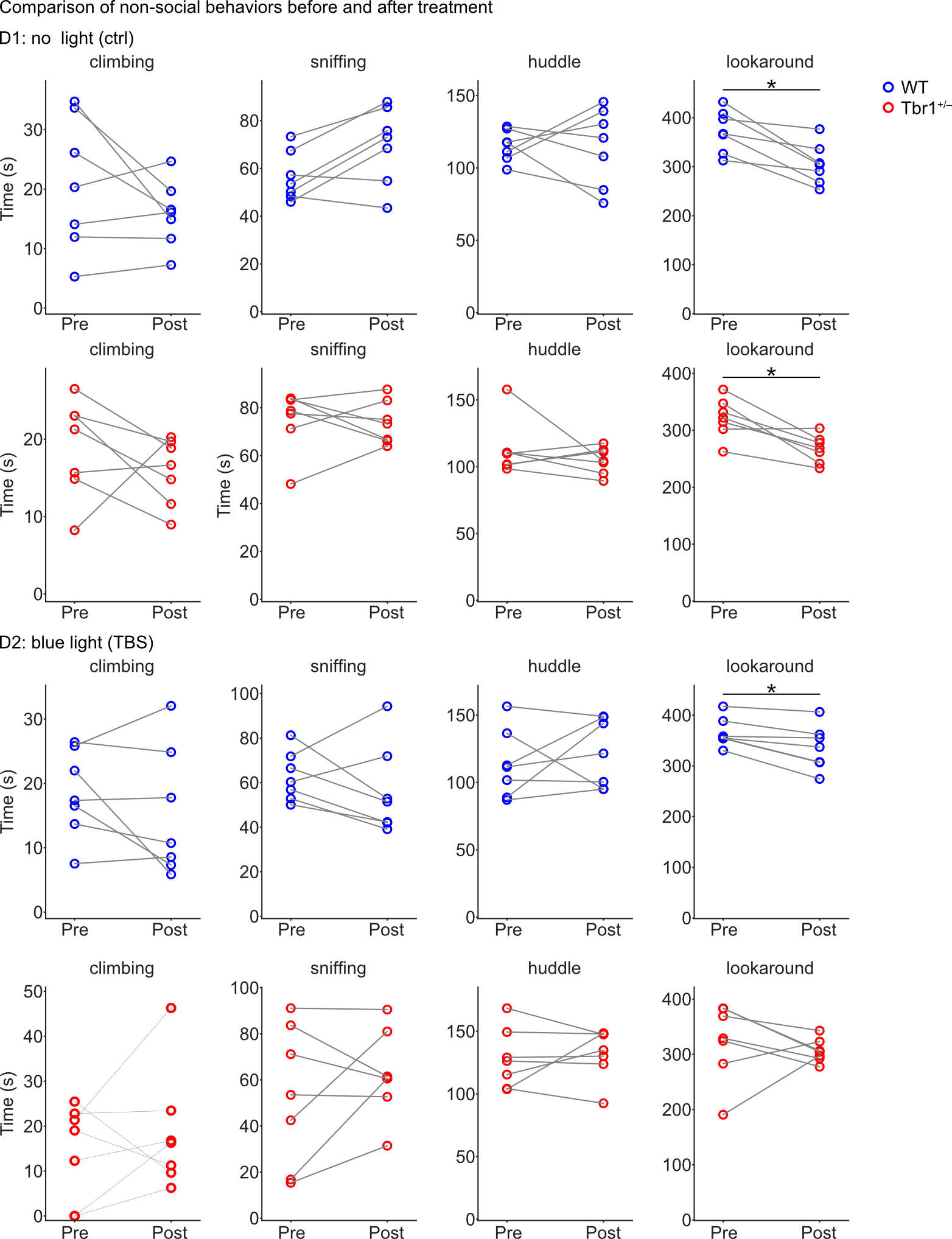

Supplement: S9 Fig — (JPG) [file pbio.3002646.s009.jpg]

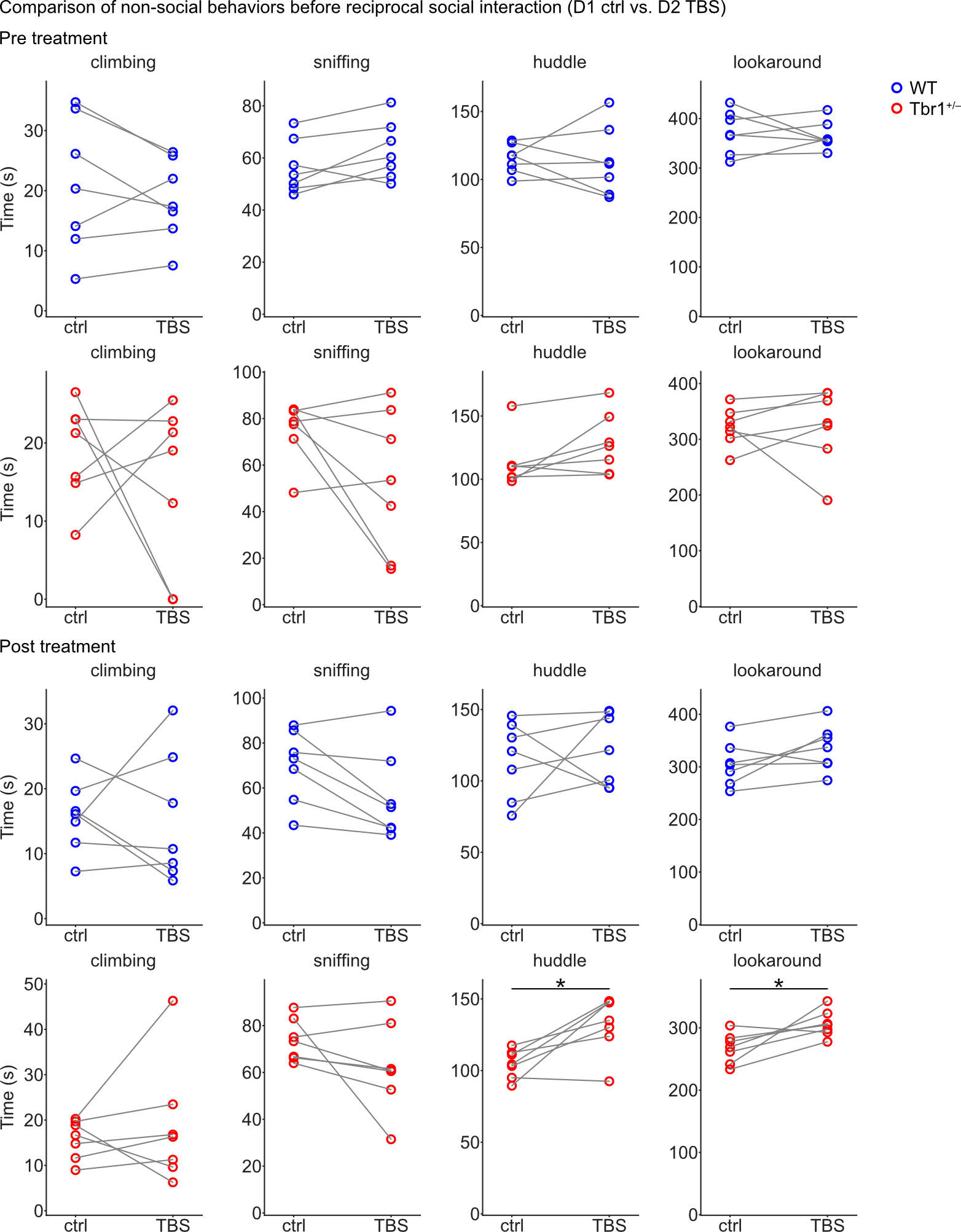

Supplement: S10 Fig — (JPG) [file pbio.3002646.s010.jpg]
