# Supplementary material for: Deep brain stimulation of the Tbr1-deficient mouse model of autism spectrum disorder at the basolateral amygdala alters amygdalar connectivity, whole-brain synchronization, and social behaviors
Source: PLoS Biol. 2024 Jul 16;22(7):e3002646. doi: 10.1371/journal.pbio.3002646 (PMC11280143; doi:10.1371/journal.pbio.3002646)
Supplement: Attachment — Submitted filename: Rebuttal_20240530.docx [file pbio.3002646.s017.docx]

**Our point-by-point response to reviewers’ comments:**

**Reviewer #1:** This manuscript investigates impaired structural and functional connectivity, aberrant whole-brain synchronization, and the impacts of deep brain stimulation at the amygdala on whole-brain synchronization and social behaviors in a Tbr1+/- autism mouse model. The authors first established a powerful whole-brain analytical platform for immunostaining and imaging, enabling the visualization and quantification of structural and functional deficits, particularly aberrant whole-brain synchronization, caused by Tbr1 haploinsufficiency. The authors further investigate the potential therapeutic implications of deep brain stimulation at the amygdala for TBR1-linked autism. They analyze how theta-burst stimulation-mediated basolateral amygdala activation may reverse the defective synchronization at a whole-brain scale caused by Tbr1 deficiency, leading to improvements in social behaviors. Their findings suggest that BLA-derived circuits play a critical role in Tbr1-linked
ASD phenotypes, highlighting the therapeutic potential of deep brain stimulation at the amygdala. Overall, the manuscript presents a stagger amount of data about the neural circuitry and network alterations underlying TBR1-linked ASD, offering novel insights into the potential therapeutic implications of deep brain stimulation at the amygdala.

I have one suggestion to improve the manuscript. It would be beneficial for the authors to discuss the duration of the behavioral changes induced by theta-burst stimulation at the basolateral amygdala and possible mechanisms underlying these changes.

**Our response to Reviewer #1:**

We very much appreciate Reviewer #1’s strong support and the specific suggestion.

Given that theta-burst stimulation is a plasticity induction protocol, we speculate that neuronal plasticity, such as long-term potentiation or long-term depression, of BLA-derived circuits is altered upon TBS at BLA. We do not know exactly how long the changes in plasticity can be maintained. However, the onset of behavioral changes after TBS of the BLA appears to be rapid because we observed differences in social behaviors when we executed the reciprocal social interaction test 10 minutes after the TBS treatment. In the future, more detailed investigations would be needed to establish how long the effect of TBS at BLA could be maintained. This information would be valuable for therapeutic purposes.

We have added this information into the revised manuscript on **page 16**.

**Reviewer #2:** The manuscript applied a whole-brain immunostaining and quantification platform to demonstrate impaired structural and functional connectivity and aberrant whole-brain synchronization in a Tbr1+/- autism mouse model. Their results emphasized the defective synchronization at a whole-brain scale caused by Tbr1 deficiency and implies a potential beneficial effect of deep brain stimulation at the amygdala for TBR1-linked autism. In addition, the current study provided the first evidence linking the DMN with the amygdala in a mouse genetic model.

Major comments:
1 The authors summed C-FOS signals across the entire LA and BLA, only the LA exhibited a difference between WT TBS and WT Ctrl. For the BLA, statistical analysis did not reveal a difference. While all injection sites were based on the Allen Mouse Common Coordinate Framework version 3 (CCFv3), the AAV injection sites covered the LA, BLAa and BLAp, which represent BLA in the previous publication (Nat Neurosci. 2014 Feb;17(2):240-7.). The C-FOS signals in BLA did not reveal a difference, the deep brain stimulation at the BLA can lead to partial improvement in social behavior. How can this result be explained? In addition, this study used unilateral stimuli for TBS. So, will bilateral stimulation enhance the effect of behavioral improvement in autism?

**Our response to Reviewer #2’s major comment 1:**

Our previous study indeed revealed contralateral innervation between two BLA in the two brain hemispheres (Huang et al., 2019, Cell Reports 29:34), as Reviewer #2 has pointed out. Moreover, Tbr1 deficiency impairs the neural activity of the BLA upon behavioral stimulation (Huang et al., 2014, Nature Neuroscience 17:240). However, the contralateral innervation of the BLA reported in our previous studies is concentrated at the anterior part of the BLA. Our current study confirms that significant ΔoChIEF between Tbr1^+/–^ and WT is present in the anterior part of the BLA (**Figure 4B, BLA middle panel, green line**). However, the anterior-posterior (AP) range of the BLA used for quantification in the current study covers the entire BLA, i.e., a much wider range than in our previous study. If we compare the same AP range as for the previous analysis, TBS at the BLA significantly elevated the C-FOS signals in the anterior BLA of WT mice (**Figure 4B, BLA bottom panel, the anterior part of the dark blue [WT_TBS] vs. light blue line [WT_ctrl]**). For Tbr1^+/–^ mice, unilateral activation at the BLA did not increase C-FOS signals at the contralateral BLA. In fact, it slightly reduced C-FOS expression in the anterior part of the contralateral BLA (**Figure 4B, BLA bottom panel, the anterior part of the red [Tbr1^+/–^_TBS] vs. salmon line [Tbr1^+/–^_ctrl]**). Thus, the response of the anterior BLA to TBS differs between WT and Tbr1^+/–^ mice. These differences between our current and previous analyses had been discussed in the first revision of our manuscript (the **last paragraph of the Discussion**, **page 17**). Based on our current study, we have also now concluded in the last sentence of the Discussion (**page 17**) that “These results highlight the sensitivity of slice-based quantifications and the necessity to analyze signals along the AP axis, a significant strength of our analytical platform.”

Based on our data, we suggest that TBS at the BLA affects other brain regions, such as DMN regions, to alter the social behaviors of Tbr1^+/–^ mice. Functional connectivity within DMN regions was partially restored upon TBS at the BLA of Tbr1^+/–^ mice (**Figure 7**). The alteration of DMN and/or other brain regions may be the circuit mechanism by which social behaviors are partially restored. We now emphasize this point on **page 17** of the second revision of our manuscript.

According to anatomical analysis, Tbr1^+/–^ mice lose a significant portion of the anterior commissure, representing an important commissure pathway connecting the two brain hemispheres. This white matter structure is also reduced or missing in patients displaying TBR1 gene deletion or mutation. It is very likely that bilateral stimulation also improves the behavioral performance of Tbr1^+/–^ mice, though we have not yet investigated that possibility. We have now added this point to our Discussion in **page 15**.

2 Figure S2 showed Spatial distribution of oChIEF-Citrine-expressing AAV injection sites in WT and Tbr1+/- mouse brains. Does the difference in injection sites affect the results of BLA circuit rewiring? 

**Our response to Reviewer #2’s major comment 2:**

BLA circuit rewiring still exists when we compare single WT and Tbr1^+/–^ brains with similar injection sites. For Reviewer #2’s information, please see the three examples presented in **Figure A** below.

Since the BLA is a complex brain region that contains diverse neuronal types, injection site variations between samples certainly contribute to the variance of labeled BLA-derived circuits. Again, see **Figure A** as examples. To account for these possibilities, we collected 13 WT and 14 Tbr1^+/–^ mouse brains and averaged YFP signals for axonal projection analysis. **Figure S2** summarizes the injection sites of all examined mice. Importantly, the results of KNN classification indicate that the spatial distributions of our injection sites were indistinguishable between the WT and Tbr1^+/–^ groups (including both control and TBS groups). Since the injection sites are indistinguishable, we were interested in comparing the wiring differences between these two sets of samples to study the impact of Tbr1 deficiency.

For Reviewer #2’s information, herein we also present an analysis restricted to the WT_TBS and Tbr1^+/–^_TBS groups (**Figure B**). Again, we found that they were indistinguishable. Thus, the impact of injection site variation on our study is limited. Inter-sample variations have been considered in our analysis of BLA circuit rewiring.

Figure A. Two-brain comparison of AAV injection sites and oChIEF patterns in WT and Tbr1^+/–^ mouse brains. One pair of examples for the injection sites at the anterior, middle and posterior parts of the BLA are shown. Right, the 3D projections of oChIEF signals. The brain samples are identical to those shown in Figure S2. Scale bar, 1 mm.

Figure B. The injection sites of WT_TBS and Tbr1^+/–^_TBS groups are indistinguishable. The same analysis as shown in Figure S2 was performed again for only the WT_TBS and Tbr1^+/–^_TBS groups.

3. The authors conducted the network analysis of functional connectivity based on inter-regional C-FOS correlations. The degree centrality (DC) and participation coefficient (PC) of individual nodes (brain regions) were calculated using NetworkX. Was this network data analysis related to real connectivity data between brain regions (according to previous literature reports), in addition to inter regional C-FOS correlations? 

**Our response to Reviewer #2’s major comment 3:**

The functional connectivity reflects the synchronization of neuronal activities between brain regions, and may not directly relate to structural connectivity. Local inhibitory microcircuits and indirect connections via multiple synaptic transmission may also contribute to neuronal synchronization. We are not sure if our whole-brain functional network analysis using C-FOS correlation is completely related to structural connectivity. However, in some subnetworks, such as the DMN in mice, it has been demonstrated previously that inter-correlated functional connectivity is related to real structural connectivity (Whitesell et al., 2021, Neuron 109:545). This is one of the reasons why we included the DMN in our analysis (the other reason is the relevance of DMN to autism). We now emphasize this point in the second revision of our manuscript (**page 17**).

4 BLA also plays a very important role in many physiological processes, such as learning and memory. The cell types of BLA are also constantly being analyzed. So, should we consider using theta burst stimulation (TBS) to regulate the specific type of neuron in BLA?

**Our response to Reviewer #2’s major comment 4:**

It is certainly worth considering cell-type issues for BLA study. Using TBS to regulate specific types of cells or specific axonal projecting neurons in the BLA would further elucidate the specific connectivities and related functions of the BLA. Different BLA connectivities may regulate different behaviors controlled by this brain structure.

In our current study, we wanted to establish all of the possible alterations to BLA circuit rewiring. Therefore, we used the pan-neuronal promoter to express oChIEF in the BLA. TBS is expected to activate all oChIEF-expressing BLA neurons. This stimulating paradigm mimics the deep brain stimulation used in human patients, a protocol that cannot target specific neuron types. Therefore, our BLA TBS can be used to preliminarily evaluate if deep brain stimulation represents a potential therapeutic approach for patients.

We have now added these points to the second revision of our manuscript (**pages 16**).

5 TBR1, a marker of glutamatergic projection neurons in the forebrain, is specifically expressed in the cerebral cortex, amygdala, olfactory bulb and hippocampus. The results showed that Tbr1+/- mice generally had more C-FOS+ cells than WT mice without any specific stimulation.  Does this mean that in some brain regions, symptoms of autism can be treated by inhibiting neuronal excitation? 

**Our response to Reviewer #2’s major comment 5:**

It is certainly possible to treat some symptoms of autism by inhibiting neuronal excitation in other brain regions. However, our data shows that reversing dyssynchronization is more critical for improving behavioral deficits. No matter whether neuronal excitation is inhibited or activated, as long as the treatment corrects the dyssynchronization of mutant brains, the behavioral impairment may be ameliorated.

**Reviewer #3:** The authors have significantly improved the presentation of their results. Much effort has been put into the figures. Some of the figures have been significantly modified, and the presentation of the results is now much clearer.  Finally, there is a new data set on the behavioral analysis of batches of mice before and after TBS. This latest set of experiments provides a concrete measure of the impact of TBS on animal behavior and, importantly, demonstrates the positive role of TBS on the social behavior of Tbr1+/- animals. 
All the changes made to this article are responding to the original comments (although I haven't found a point-by-point response letter) and even go beyond the original requests with the addition of behavioral analysis. 

Minor comment: I am surprised that the striatum is not among the regions listed given the severe neocortical defects observed in Tbr1-/- mutants. Indeed, cortico-striatal connections are increased in these animals, and I would have expected the striatum to be similarly affected in Tbr1+/- heterozygous animals.

**Our response to Reviewer #3’s minor comment:**

In the current study, we did not investigate cortical-striatal connections in Tbr1^+/–^ mice. We examined only the structural and functional connectivities of the BLA. We did detect rewired BLA axonal projections to the CP (the major brain region of the striatum) in Tbr1^+/–^ mice (**Table S1**). Tbr1 deficiency impaired axonal projections to the contralateral CP, but intensified axonal projections to the ipsilateral CP (**Table S1**). For functional connectivity, C-FOS expression in the CP of the Tbr1^+/–^ ctrl group was higher than that of the WT ctrl group. TBS at the BLA exhibited the opposite effects on C-FOS expression for the CP when comparing the WT and Tbr1^+/–^ groups (**Table S1**). For Reviewer #3’s information, we now highlight the CP in **Figure C**.

Figure C. The CP is highlighted for all oChIEF and C-FOS analyses in the current study.
